# Mechanisms and Consequences of Developmental Acceleration in Tadpoles Responding to Pond Drying

**DOI:** 10.1371/journal.pone.0084266

**Published:** 2013-12-16

**Authors:** Ivan Gomez-Mestre, Saurabh Kulkarni, Daniel R. Buchholz

**Affiliations:** 1 Ecology, Evolution, and Development Group, Doñana Biological Station, Consejo Superior de Investigaciones Científicas, Seville, Spain; 2 Department of Biological Sciences, University of Cincinnati, Cincinnati, Ohio, United States of America; 3 Department of Pediatrics, Yale School of Medicine, Yale University, New Haven, Connecticut, United States of America; University of Sao Paulo, Brazil

## Abstract

Many amphibian species exploit temporary or even ephemeral aquatic habitats for reproduction by maximising larval growth under benign conditions but accelerating development to rapidly undergo metamorphosis when at risk of desiccation from pond drying. Here we determine mechanisms enabling developmental acceleration in response to decreased water levels in western spadefoot toad tadpoles (*Pelobates cultripes*), a species with long larval periods and large size at metamorphosis but with a high degree of developmental plasticity. We found that *P. cultripes* tadpoles can shorten their larval period by an average of 30% in response to reduced water levels. We show that such developmental acceleration was achieved via increased endogenous levels of corticosterone and thyroid hormone, which act synergistically to achieve metamorphosis, and also by increased expression of the thyroid hormone receptor TRΒ, which increases tissue sensitivity and responsivity to thyroid hormone. However, developmental acceleration had morphological and physiological consequences. In addition to resulting in smaller juveniles with proportionately shorter limbs, tadpoles exposed to decreased water levels incurred oxidative stress, indicated by increased activity of the antioxidant enzymes catalase, superoxide dismutase, and gluthatione peroxidase. Such increases were apparently sufficient to neutralise the oxidative damage caused by presumed increased metabolic activity. Thus, developmental acceleration allows spadefoot toad tadpoles to evade drying ponds, but it comes at the expense of reduced size at metamorphosis and increased oxidative stress.

## Introduction

Adaptive developmental plasticity evolves in response to environmental heterogeneity when organisms can unambiguously perceive the environment through reliable cues, and there exists a cross-environmental trade-off such that no single phenotype can maximise fitness across environments [1,2]. Plasticity therefore allows organisms to occupy fluctuating environments by developing appropriate matching phenotypes [3]. 

Amphibians are the group of terrestrial vertebrates with the greatest diversity of life histories, but the majority maintain an aquatic larval stage that metamorphoses into a terrestrial juvenile [4,5]. Although some species breed in permanent water bodies such as rivers, lakes, and marshes, most species depend upon temporary water bodies for larval development, from temporary ponds to ephemeral rain pools, rock crevices, or water pockets on vegetation like bromeliads or bamboo stumps. Throughout their evolutionary history, amphibian larvae have excelled at exploiting such temporary aquatic systems because they often show a high degree of developmental plasticity enabling them to decouple growth and differentiation to a remarkable extent [6]. Under abundant food availability, low risk of predation, and low risk of desiccation, amphibian larvae are capable of growing while only slowly progressing in (or arresting) development, but they accelerate development at the expense of continued growth when adverse conditions are met, triggering an early metamorphosis [7,8]. Chief among larval risks in temporary systems is pond drying, which can be detected by tadpoles in the form of shallow water and proximity to the water surface (i.e. water column height; [9]). Environmental assessment by amphibian larvae may be so fine-grained that developmental acceleration can even decelerate if the conditions in the aquatic system ameliorate [9,10]. Developmental acceleration in response to drying of the aquatic habitat has long been reported in anurans [8,11,12] and has been found in nearly all species examined [4,13,14]. Accelerated metamorphosis, however, cannot occur prior to achieving an apparent developmental threshold [15,16]. 

This ability to adjust larval period to the local aquatic habitat quality and duration may have played a major role in allowing different populations and even species to adapt to widely divergent environments. As a result, divergent reaction norms among lineages adapted to aquatic habitats of different flooding regime have been reported below and above the species level [17-19]. This suggests that ancestral plasticity may have allowed differences among lineages to evolve by genetic accommodation, an idea supported by the fact that morphological consequences of acceleration observed within populations are also mirrored among populations and species with adaptively divergent larval periods [20,21]. Thus, tadpoles reared in conditions that induce fast development result in juveniles with reduced limb length compared to siblings reared under control conditions, and these differences scale up among populations and even species so that lineages with faster developmental rates result in shorter-legged individuals [20,21]. 

Tadpoles’ capacity to adaptively tune the time to metamorphosis to local conditions is controlled by their neuroendocrine system. In response to specific environmental stimuli during pond drying, the hypothalamus increases production of corticotropin-releasing-hormone (CRH) that stimulates the production of pituitary hormones that activate the thyroid and interrenal glands in all anurans and non-neotenic urodeles examined [22,23]. The activation of these hypothalamo-pituitary-thyroid (HPT) and hypothalamo-pituitary-interrenal (HPI) axes results in increased levels of thyroid hormone (TH) and corticosterone (CORT) [24-26]. While TH is the primary morphogen, CORT synergises with TH to enhance the sensitivity of tissues to TH through the upregulation of TH receptors and monodeiodinase enzymes in specific tissues [25,27]. Thus, the shorter larval periods observed under risk of pond drying are mediated by increased production of TH and CORT via the HPT and HPI axes causing developmental acceleration [25]. What is not clear is the extent to which hormones and their receptors are increased throughout development within individuals reared in conditions favouring rapid metamorphosis.

Despite the advantages of accelerated metamorphosis, developmental acceleration comes at the cost of physiological, morphological, and life-history consequences. At the level of physiology, developmental acceleration seems to be a rather costly effort consuming a large fraction of the fat bodies accumulated during larval growth and/or preventing their accumulation [28]. Furthermore, developmental acceleration reduces size at metamorphosis [8,28,29], which is a commonly observed major factor influencing juvenile survival [30,31]. Accelerated metamorphosis also decreases size at first reproduction [32], challenges immune response of postmetamorphic individuals [33], and affects juvenile morphology, resulting in shorter-limbed metamorphs [6,34]. Such fat burning and sustained physiological effort would be expected to alter the redox balance, greatly increasing the production of reactive oxygen species (ROS). ROS are normally produced during development and may even play a signalling role as secondary messengers in development [35,36]. However, when the production of ROS causes a redox imbalance oxidative stress occurs, and if ROS production exceeds the organism’s antioxidant capacity, it results in oxidative damage [37,38]. Sustained developmental acceleration in response to pond drying may cause increased ROS production and hence may require amphibian larvae to increase antioxidant enzymatic activity.

Our aims for this study were to examine the main endocrine mechanisms thought to regulate developmental acceleration in tadpoles and to quantify whether tadpoles incur oxidative stress during acceleration. Thus, we exposed tadpoles of the highly developmentally plastic Western spadefoot toad (*Pelobates cultripes*) to reduced water levels simulating pond drying, and analysed their developmental response. We quantified tissue content of thyroid hormone and blood corticosterone concentrations, which have been shown separately to play a role in accelerated development in other species. We also tested the prediction that thyroid hormone receptors (TRβ) would be up-regulated in response to pond drying as a measure of increased hormone functionality. Finally, we evaluated the oxidative stress caused by accelerated development measuring the activity of three antioxidant enzymes and a biochemical marker of oxidative damage. 

## Materials and Methods

### Ethics Statement

Egg clutches of the western spadefoot toad *Pelobates cultripes* were collected within the Biological Reserve of Doñana National Park with collecting permits granted by Consejería de Medio Ambiente from Junta de Andalucía. The experimental procedures and euthanasia of tadpoles were conducted at Estación Biológica de Doñana, CISC, following protocol ‘12_53-Gomez’ approved by the Institutional Animal Care and Use Committee (IACUC) at Estación Biológica de Doñana.

### Experimental setup

In March 2009, we collected portions of approximately 50 eggs from each of six Western spadefoot toad clutches from two different ponds at the Doñana Biological Reserve, within the Doñana National Park (Huelva, southwestern Spain). *Pelobates cultripes* tadpoles are large and have a long larval period (usually between 84 and 130 days; [20,39]. Paradoxically, ecological and mechanistic aspects of developmental plasticity have been studied in other spadefoot toad species [11,40,41], but those species have a much more canalised development and consequently reduced levels of plasticity compared to *P. cultripes* [20,28]. 

**Figure 1:**
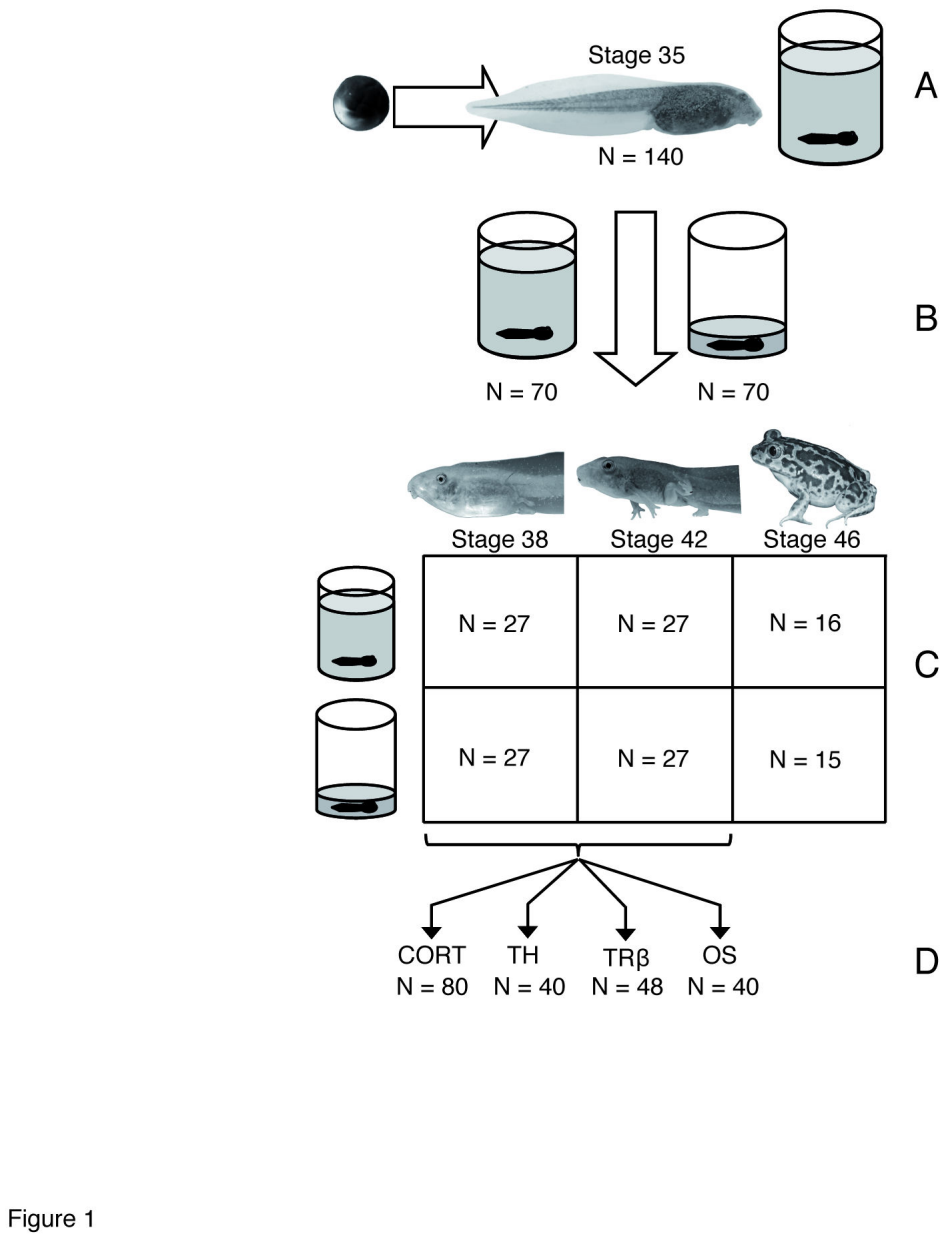
Experimental design and sample distribution. (A) Field collected eggs were brought into the laboratory. Once they reached the free feeding stage (Gonser stage 25), 140 tadpoles were individually raised in 3 L plastic containers until Gosner stage 35. (B) As tadpoles reached stage 35, they were assigned to either constant water volume (3L), or reduced water volume (350 mL). (C) Tadpoles were then raised up to predetermined stages: Gosner stages 38 and 42 (stages at which the physiological parameters were determined), or until metamorphosis was complete (Gosner stage 46). (D) Corticosterone (CORT) was determined by electroimmuno assays from plasma samples obtained from 80 tadpoles across developmental stages and water treatments. Tail tissue from those tadpoles was used for thyroid hormone (TH) radioimmuno assays and determination of the level of expression of the thyroid hormone receptor TRβ via qPCR. Levels of oxidative stress and activity of antioxidant enzymes were determined after whole body homogenization.

The eggs were brought into a climatic chamber at Doñana Biological Station and allowed to hatch in shallow trays with aerated carbon-filtered tap water. Once larvae reached the free-feeding developmental stage, i.e. Gosner stage 25 [42], we randomly placed 140 tadpoles individually in 3 L clear plastic buckets (168 mm in diameter x 184 mm tall). The chamber was set up at constant 24 °C and a 12:12 photoperiod, water was renewed twice a week, and tadpoles were fed *ad libitum* rabbit chow, ca. 1 g/week depending on stage and size. We raised all tadpoles similarly (Figure 1A) until Gosner stage 35, a mid-prometamorphosis stage at which they show their maximum capacity for developmental acceleration [28], and then reduced the water volume on half of the experimental units to trigger acceleration (Figure 1B). Tadpoles in the high water volume were kept in 3 L of water whereas those in the low water treatment had only ca. 350 mL, a water column 20 mm high that was just enough to cover the largest tadpoles [28]. Tadpoles in each water level were raised to one of two predetermined developmental stages (Figure 1C), either Gosner stage 38, in late prometamorphosis when larvae already have well developed hind limbs and the inner metatarsal tubercule is formed; or Gosner stage 42, right at metamorphic climax when forelimbs emerge and just prior to initiation of tail resorption [42]. Hence the design consisted of two water levels (high vs. low) x two developmental stages (Gosner 38 vs. 42), with 27 replicates per water level x stage combination. Shelves in the climatic chamber were distributed at three heights and hence potentially experiencing a temperature gradient, so each shelf was considered a block, and each block held 9 replicates per treatment. For each tadpole, we recorded the date at which they reached Gosner stage 35, 38, and 42, and we weighed them after blotting dry. We estimated growth rate as log(mass) – log(larval period). In addition, we raised 15 extra individual tadpoles to the completion of tail resorption (Gosner stage 46) in each water level treatment to assess the effect of pond drying on postmetamorphic morphology (Figure 1C). We used callipers to measure snout-to-vent length and hindlimb length to the nearest 0.1 mm. We took two measurements of each trait to estimate repeatability, and averaged them for analysis.

### Hormone measurements

We determined the level of corticosterone through enzyme immunoassays (EIAs) conducted on plasma samples. A total of 20 tadpoles per treatment (i.e., two developmental stages x two water volumes, total *N* = 80, Figure 1D) were assayed for corticosterone determinations. Tadpoles at the appropriate developmental stage (either 38 or 42 Gosner stage) were deeply anesthetised by immersion in MS-222, and then placed under a dissecting stereoscope (Discovery V8, Zeiss, Norway) and operated at a 20X magnification. We cut and pulled apart the ventral skin to access the pericardium. Then we dried up the liquid of the abdominal cavity with filter paper and sectioned the aorta, rapidly collecting the blood spillage with a 30-gauge needle mounted on a 1mL syringe, and pouring the blood in heparinised tubes kept at 4 °C over ice. This procedure was conducted under 3 min and yielded ~80 µL of blood per individual. After blood extraction, the animal was euthanized with MS-222, and then eviscerated, snap frozen in liquid nitrogen, and preserved at -80 °C for determination of TH, TRβ, and oxidative stress enzymes. Blood samples were then centrifuged at 4000 g for 20 min, and the supernatant was stored at -80 °C in fresh Eppendorf tubes for later analyses. Samples were assayed with a corticosterone EIA kit (#500655, Cayman Chemical, MI, USA), using the competition between corticosterone and a corticoterone-acetylcholinesterase conjugate used as tracer. The lower detection limit for this assay was 16 pg/mL. After incubation of the sample and the two standard curves with tracer and antiserum, the plates were washed and developed with Ellman’s reagent following the manufacturer’s indications. We then read the plates on a Victor 3 1420 (Perkin-Elmer, MA, USA) at 405 nm and concentrations were estimated from interpolation to the standard curves using a four-parameter fit. Average coefficient of variation for sample duplicates was 16.91 %. 

We determined tissue content of T4 (thyroxine, the precursor of T3 or tri-iodothyronine, the active form of TH) from tail samples by radio-immunoassay (RIA). Tail tissue instead of plasma was used in this case because the blood samples obtained were insufficient to conduct RIA. We used a sample size of 10 tadpoles per developmental stage and water level (total *N* = 40, Figure 1D). We extracted TH from tail tissue, homogenising the tissue, centrifuging and extracting the hormone from the supernatant with chloroform and ion-exchange chromatography prior to RIA following previous protocols [43,44]. Lower detection limit was 0.15 pg/mL/mg and average coefficient of variation was 8.20 %.

### RNA isolation, cDNA synthesis, quantitative PCR

TRβ gene expression measurements were carried out as done previously [45]. Briefly, total RNA was extracted from frozen samples using Trizol reagent following the manufacturer's protocol (Invitrogen), and cDNA was synthesized using 2 ug of total RNA using the High Capacity cDNA Reverse Transcription Kit following the manufacturer's protocol (Applied Biosystems). Quantitative PCR (qPCR) for TRβ and the housekeeping gene rpL8 (2uL of neat or 10-fold diluted cDNA respectively) was carried out in single-plex reactions with Taqman FAM-labeled probes in Universal PCR Master Mix using a 7300 Real Time PCR System (Applied Biosystems). Primer-probe sets were designed previously [45]: TRβ: forward primer 5' GGAACCAGTGCCAAGAATG, reverse primer 5' TCATCCAAGACCAAGTCTGTTG, probe 5' CGCTTCAAAAAGTG, rpL8: forward primer 5' CACAATCCTGAAACCAAGAAAACCA, reverse primer 5' CCACACCACGGACACGT, probe 5' AAGGCCAAGAGAAACT. No template controls were used and failed to detect any reaction product contamination. We estimated TRβ gene expression from 12 tadpoles per stage and water level (Figure 1D). All samples were run in duplicate, and averages were used for subsequent analyses. Average coefficient of variation for sample duplicates was 8.88 %.

### Oxidative stress enzyme activity

In a subset of animals (N = 40, 10 tadpoles per stage and water level, Figure 1D), we measured activity of three enzymes involved in protection against various important ROS such as superoxide anions and hydrogen peroxide [38,46]: superoxide dismutase (SOD), catalase (CAT), and glutathione peroxidase (GPx). As an indication of oxidative damage, we quantified thiobarbituric acid reactive substances (TBARs) formed mostly during lipid peroxidation [47], namely lipid hydroperoxides and aldehydes [48]. Frozen individuals were homogenized with a Miccra (Miccra D-1) homogeniser in a buffered solution (100 mM Tris-HCl with 0.1 mM EDTA, 0.1% triton X-100, pH 7.8 and 0.1 mM PMSF; 1:4, w:v) to inhibit proteolysis. We then centrifuged the samples at 4000 g for 30 min at 4° C. Total protein content in the supernatant fluid was determined following a standard Bradford’s procedure [49]. Enzyme activity was determined colorimetrically. We determined catalase activity using potassium permanganate (KMnO_4_) as an oxidizing and coloring agent following [50]. KMnO_4_ reacts with hydrogen peroxide, the catalase substrate, and is hence reduced producing a red product. We read absorbance at a wavelength of 480 nm five minutes after KMnO_4_ was added to the samples. We prepared standard curves of commercial catalase (SIGMA-60634) and expressed the catalase activity as U / mg of total proteins. Average coefficient of variation for sample duplicates was 5.14 %, and the lower detection threshold was 125 U/mL.

Similarly, we determined the activity of SOD indirectly measuring the inhibition rate of cytochrome C reduction. Superoxide radicals (O_2_^-^) oxidize cytochrome C except in the presence of SOD, which competes for O_2_^-^ generated by hypoxanthine and xanthine oxidase action, reducing cytochrome C and producing hydrogen peroxide (H_2_O_2_) and oxygen. We monitored the increase in absorbance at 550 nm and defined one unit of SOD as the amount of enzyme that inhibited the rate of reduction of cytochrome C by 50% at 25 °C following [51]. Lower detection limit for SOD was 1 U /mg protein, and the average coefficient of variation for duplicate samples was 8.17 %. We determined glutathione peroxidase (GPx) activity following Paglia and Valentine [52]. Oxidised glutathione (GSSG) is continually reduced due to an excess of glutathione reductase (GR) and produces a constant level of reduced glutathione (GSH). Production of GSH from oxidised glutathione requires NADPH, and we monitored NADPH oxidation reading absorbance at a wavelength of 340 nm. Lower detection limit for GPx was 2 U/mg protein, and the average coefficient of variation for sample duplicates was 10.88 %. Finally, we measured lipid peroxidation following Buege and Aust [53]. Lipid peroxidation produces, among other compounds, malondialdehyde (MDA). MDA reacts with acid to give a thiobarbituric acid reactive substance (TBARS), a red product absorbing at 535 nm. To obtain TBARS concentrations we measured the optical density values for the blank and for the calibration curve. We calculated the TBARS concentration (in nmolTBARS/ml) from the absorbance of each sample, subtracting the blank values and comparing with the calibration values. Lower detection limit for TBARS was 0.1 µM, and the average coefficient of variation for sample duplicates was 3.54 %.

### Statistical analyses

All analyses consisted of generalised linear models fitted using the Glimmix procedure (SAS Institute, Cary, NC, USA). Survival data was modelled assuming a binomial distribution with a logit link function. All other variables (larval period, size at metamorphosis, corticosterone, TH, TRΒ, CAT, SOD, GPx, and TBARs) were modelled either using a Gaussian or a gamma error distribution (with identity or log link functions, respectively), chosen on the basis of the lowest Akaike Information Criterion (AICc) observed in alternative models. Expression of a housekeeping gene, rpL8, was introduced in the model as a covariate when analysing differences among treatments in TRβ. Experimental block was introduced in the analyses as a random factor. Analysis of relative limb length included snout-vent-length as a covariate in the model, and adjusted means were calculated for mean hindlimb length in each treatment controlling for differences in SVL. 

## Results

### Developmental acceleration in response to decreased water levels

**Figure 2:**
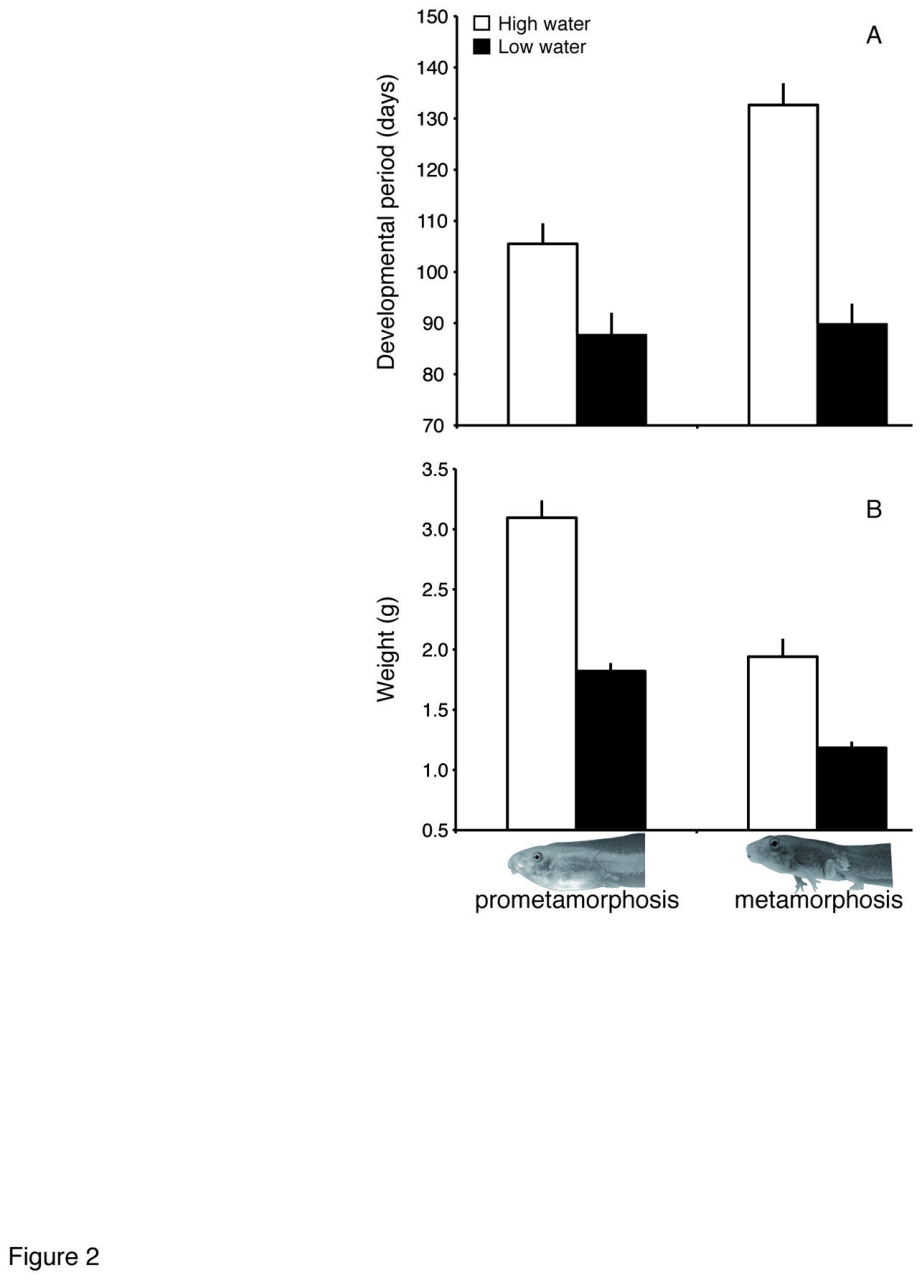
Developmental responses to decreased water levels. (A) Western spadefoot toad tadpoles accelerated development (required a shorter time to reach the target developmental stage) when water level was experimentally decreased. (B) Faster development resulted in smaller animals at each developmental stage. Bars indicate mean values + SE.

**Figure 3:**
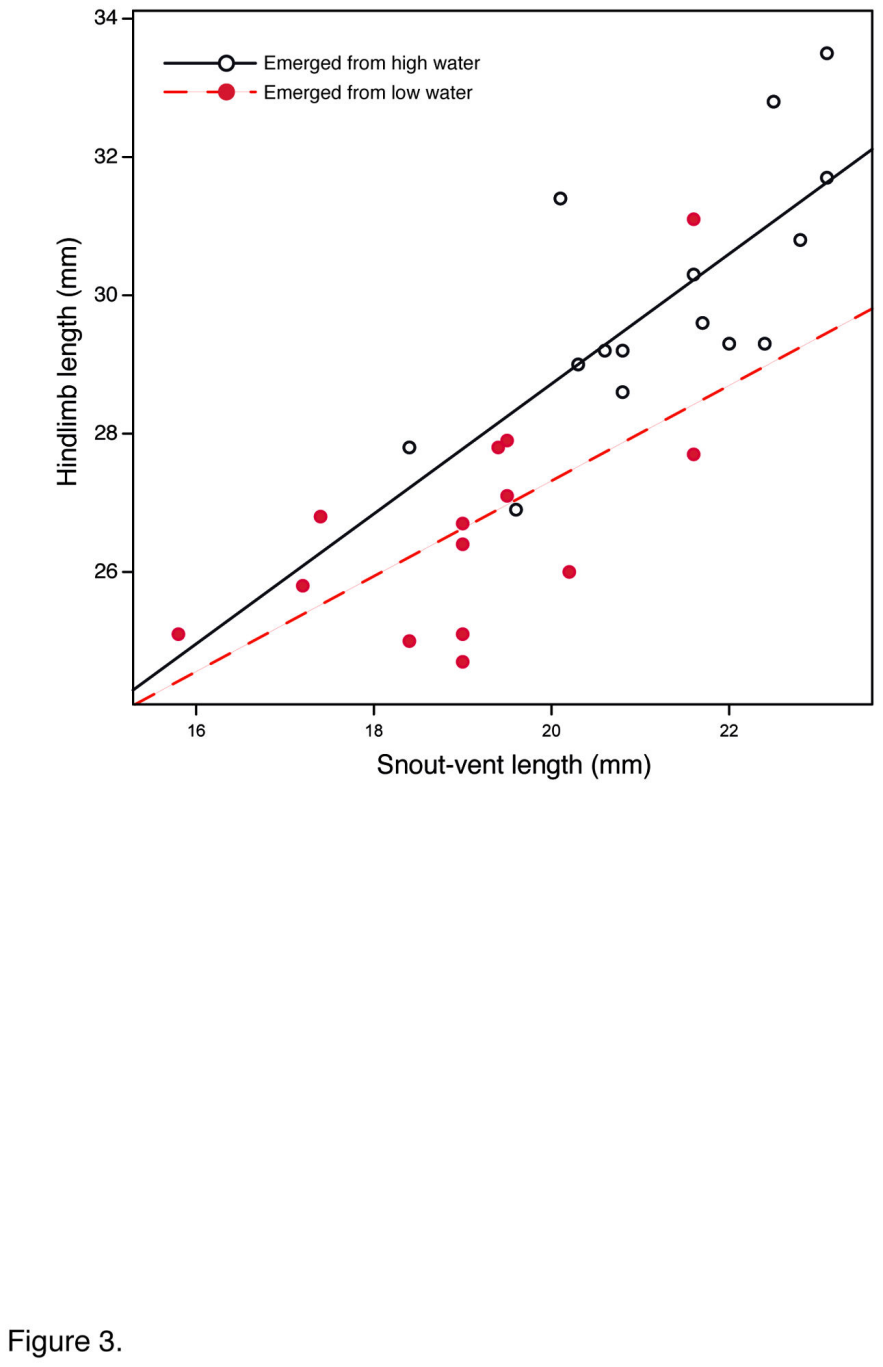
Spadefoot toad juveniles emerging from reduced water treatment were smaller and had proportionately shorter hind legs.

Decreased water levels reduced tadpole survival from 94.2% to 79.3%, indicating that it was a considerable source of stress (df=1,99, χ^2^ = 4.19, *P* = 0.041). Tadpoles in full water volume took on average 105.5 days (± 4.0 SE) to reach Gosner stage 38 and 132.65 days (± 4.25) to reach metamorphosis (Gosner stage 42), whereas tadpoles exposed to low water volume took 86.38 days (± 4.43) to reach stage 38 and 89.77 (± 4.0) days to reach metamorphosis, resulting in highly significant reductions in larval period (*F*_1,44_ = 23.20, *P* < 0.0001 for days to Gosner stage 38; *F*_1,45_ = 40.91, *P*<0.0001 for days to Gosner stage 42) (Figure 2A). Counting from the moment when water volume was reduced, development was accelerated by 32.3% on average in response to decreased water level. As expected, body mass at metamorphosis was significantly lower in tadpoles exposed to low water (3.095 ± 0.107 vs. 1.82 ± 0.122 at Gosner stage 38 and 1.940 ± 0.114 vs. 1.183 ± 0.119 at Gosner stage 42; *F*_1,88_ = 90.78, *P* < 0.0001) (Figure 2B). Growth rate was also lower for animals exposed to low water (*F*_1,85_ = 29.68, *P* < 0.0001), and juveniles emerging from the low water treatment had on average 5% shorter hind limbs (27.572 ± 0.398 mm in juveniles from low water vs. 29.045 ± 0.387 mm in juveniles from high water, adjusted means; *F*_1,26_ = 5.66, *P* = 0.025) (Figure 3).

### Mechanisms mediating developmental acceleration

**Figure 4:**
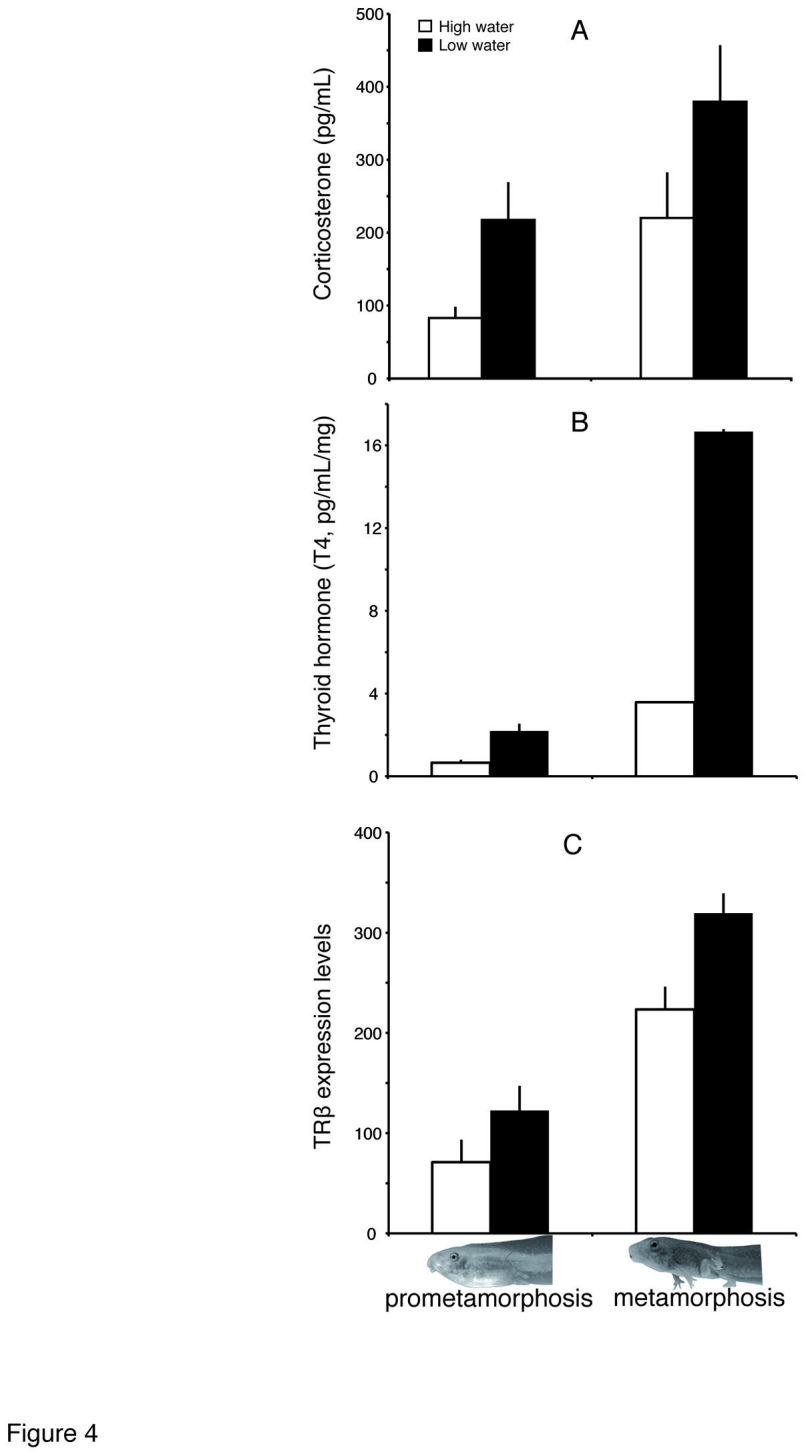
Mechanisms of tadpole developmental acceleration. Levels of corticosterone (A), thyroid hormone (B), and expression of thyroid hormone receptor TRβ (C) were higher for tadpoles exposed to decreased water levels (black bars) than for those in constant water (white bars). Error bars indicate + SE.

Corticosterone (CORT) concentration increased from prometamorphosis (Gosner stage 38) to metamorphic climax (Gosner stage 42) (*F*_1,55_ = 10.34, *P* = 0.002). However, CORT in stage-matched tadpoles was higher in those exposed to low water levels (*F*_1,55_ = 10.0, *P* = 0.003) (Figure 4A) by 2.61 fold at Gosner stage 38 and 1.72 fold at Gosner stage 42. However, we found no significant interaction between water level and developmental stage in CORT levels.

Thyroid hormone was also higher at metamorphic climax than at pre-metamorphosis (*F*_1,35_ = 120.34; *P* < 0.0001), and tadpoles exposed to low water had higher TH tissue content than those in full water volume (*F*_1,35_ = 64.84; *P* < 0.0001) (Figure 4B). The average fold increase in TH concentration was 3.31x at Gosner stage 38 and 4.65x at Gosner stage 42. We found no significant water level by stage interaction. In addition to increased TH levels, our quantitative PCR results showed a significant increase in the expression of the TH receptor (TRβ) in response to low water level (*F*_1,46_ = 10.69, *P* = 0.002) (Figure 4C), controlling for the expression of a housekeeping gene, rpL8 (see Materials and Methods). Tadpoles in low water experienced a 1.74 fold increase in TRβ at Gosner stage 38, and a 1.43 fold increase with respect to tadpoles in high water at metamorphic climax. We found no significant interaction between water level and developmental stage in TRβ expression.

### Oxidative stress as a consequence of developmental acceleration

**Figure 5:**
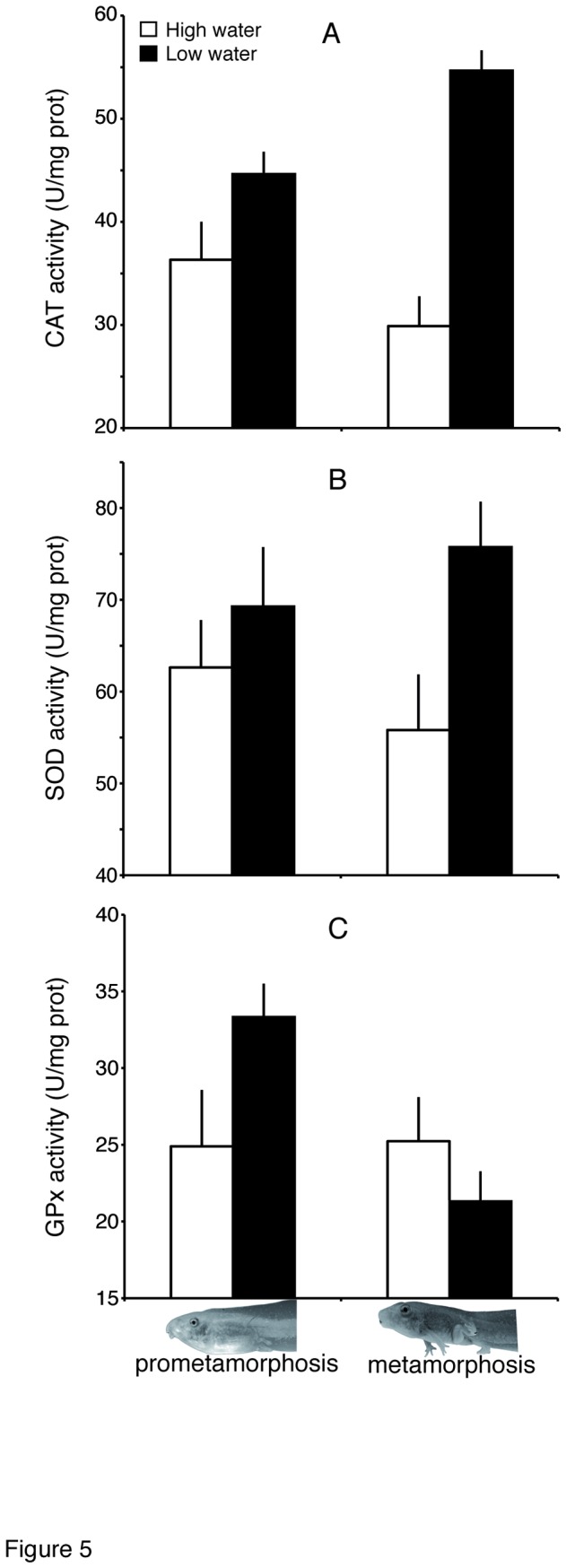
Activity of three antioxidant enzymes in tadpoles exposed to decreased water level compared to those kept in constant water level. Catalase (A) and superoxide dismutase (B) activity increased in tadpoles experiencing decreased water level, in both developmental stages. Glutathione peroxidase (C) also increased when tadpoles faced low water level, but only in prometamorphosis.

Tadpoles exposed to low water levels showed higher levels of catalase activity (*F*_1,34_ = 9.44, *P* = 0.004) than tadpoles kept in full water volume (Figure 5). The observed activity was on average 18.6% higher in low water tadpoles at Gosner stage 38, and 54.67% higher at Gosner stage 42. Similarly, we also found higher levels of superoxide dismutase activity in tadpoles exposed to low water level (*F*_1,34_ = 5.45, *P* = 0.026) (Figure 5). SOD activity was 9.61% higher in low water tadpoles at Gosner stage 38, and 26.31% higher at Gosner stage 42. No significant interaction between water level and developmental stage was found for these two enzymes. Glutathione peroxidase (GPx), however, was slightly higher in low water tadpoles, but only at stage 38 hence resulting in a small but significant interaction between water level and developmental stage (*F*_1, 34_ = 4.44, *P* = 0.043). Reduced water volume, however, did not affect levels of TBARs (*F*_1, 34_ = 0.00, *P* = 0.971), but changed instead with developmental stage (*F*_1, 34_ = 50.51, *P* < 0.0001) so that animals at metamorphic climax (Gosner stage 42) showed a 1.95-fold increase in TBARs values (Gosner38: 4.64 ± 0.55; Gosner 42: 9.03 ± 0.52). No significant interaction between water level and stage was found.

## Discussion

### Mechanisms of developmental acceleration

Tadpoles of the Western spadefoot toad accelerated development by an average of 32% in response to decreased water levels in our experiment resulting in a shorter larval period by over 2 weeks (Figure 2), which is among the highest responses reported in the literature for anurans [4,54]. Interestingly, the magnitude of this response from this population of southern Spain matches the whole range of variation reported in the literature for larval period in this species [20,39], which also encompasses geographic variation among populations. 

Consistent with previous studies, our results show that developmental acceleration in response to pond drying is accompanied by increased levels of TH and CORT (Figure 4), presumably driven by increased CRH [22,25]. TH and CORT synergize to increase the rate of metamorphic change [55], and thus, the higher levels of both hormones in low water contribute to accelerated development. Moreover, because TRβ is a th direct response gene [56], the up-regulation of TRβ expression in low water was consistent with increased TH production. The resulting increased tissue sensitivity and responsivity to circulating TH from higher TRβ expression thus also contributed to increased rate of metamorphosis in low water levels [45,57].

Our previous phylogenetic and comparative experimental work showing how changes in developmental rate among species mirrored differences among individuals reared in different conditions led us to suggest that life-history evolution in spadefoot toads may have resulted from genetic accommodation of ancestral plasticity into the canalised fast development of some species [20,28]. To further substantiate this model, we predicted that among species differences in larval period and physiological and genetic parameters regulating it (i.e., hormone titre, TRβ expression level) would mirror ancestral within-species variation. Importantly, *P*. *cultripes* represents the ancestral state of developmental plasticity in spadefoot toads [20]. Here, we found that increased TH and CORT levels are maintained at higher levels throughout development in response to low water in *P. cultripes*, and these results mirror fixed differences in regulatory mechanisms found among spadefoot toad species. Specifically, higher TH content and higher TR expression across development is observed in species with short larval periods (*Scaphiopus couchii*) compared to other spadefoot species reared under identical conditions and in *P. cultripes* reared at low water compared to *P. cultripes* reared at high water [45,58]. In the case of TR expression, *S. couchii* has constitutively higher levels of TRα rather than TRβ, but the end result of increased TR-mediated tissue sensitivity to TH is apparently the same [45]. Thus, these data support our predictions about how short larval periods with low plasticity may have evolved from longer, plastic larval periods via genetic accommodation. 

### Costs and consequences of developmental acceleration

Developmental acceleration in Western spadefoot toad tadpoles resulted in smaller, shorter-limbed juveniles, as reported for other species [54,59,60] and predicted in models of anuran developmental responses to larval growing conditions [6]. Such reduction in size at metamorphosis is expected to have a plethora of consequences for juvenile survival, age of first reproduction, and fecundity that could even scale-up to demographic consequences [32,61]. 

Undergoing metamorphosis seems to be metabolically intensive, and environmentally altered metabolic activity may carry some consequences. A small fraction of the reactive oxygen species (ROS) generated in the organism is controlled and has a role in cell signalling [36]. However, most ROS (about 90%) are generated as by-products of metabolic activity [62,63]. If not neutralised, ROS cause oxidative damage to many key biomolecules, including DNA (especially mitochondrial DNA and telomeres), proteins, and lipids [62-64]. Such damage has the potential to influence life histories over both short and long timescales, including accelerated senescence as unrepaired cell damage accumulates [38,63], but also as a physiological cost of reproduction, immune function, or general metabolic activity [37].

We detected increased lipid peroxidation (i.e. increased TBARs) in tadpoles at metamorphic climax regardless of experimental treatment. ROS production and antioxidant activity is known to vary with developmental stage [65], and the differences observed across development may reflect at least in part changes in signalling pathways. Here we found that environmentally induced developmental acceleration caused oxidative stress in spadefoot toad tadpoles, as it significantly increased activity of antioxidant enzymes. Oxidative stress may be an inescapable consequence of developmental acceleration in tadpoles partly because of enhanced physiological and metabolic effort, but also because sustained high glucocorticoid production is a direct cause of oxidative stress in vertebrates [66] and increased CORT is key in accelerating metamorphosis. However, we found no evidence for increased oxidative damage (presence of lipid hydroperoxides and aldehydes) with decreased water level. These results suggest that increased antioxidant activity may have been sufficient to neutralise oxidative damage in our experiment. Whether genotypes or lineages adapted to fluctuating environments, and hence more developmentally plastic, are better at coping with oxidative stress than less plastic ones is something that will require further study.

Increased oxidative stress may be a key determinant in linking accelerated development to increased metabolism, reduced longevity, and delayed age of sexual maturation. Similarly [46], showed increased activity of antioxidant enzymes during phases of compensatory growth in a damselfly following transient periods of starvation. Moreover, damselflies forced to undergo compensatory growth incur physiological costs that include increased metabolic rate and energy storage depletion [67]. In mammals, increased growth hormone (GH) increases growth rate but at the expense of increasing the production of superoxide radicals [68], and growth rate is positively associated to oxygen consumption and ROS production in transgenic strains of zebrafish [69]. Developmental acceleration and compensatory growth hence seem to come at comparable physiological costs among different animal groups. Organisms with a large degree of decoupling between growth and differentiation can grow quickly but not progress in developmental stages or develop quickly with little or no growth occurring [6]. However, both accelerated growth and accelerated development seem to incur similar metabolic and physiological costs, perhaps mediated through oxidative stress. Oxidative stress could thus be a common cause of reduced longevity and other long-term deleterious effects of rapid growth [63] and rapid development. We are unaware of evidence for long-term consequences of oxidative stress in amphibians, which will require further study.
